# Image-guided hepatopancreatobiliary surgery using near-infrared fluorescent light

**DOI:** 10.1007/s00534-012-0534-6

**Published:** 2012-07-13

**Authors:** Floris P. R. Verbeek, Joost R. van der Vorst, Boudewijn E. Schaafsma, Merlijn Hutteman, Bert A. Bonsing, Fijs W. B. van Leeuwen, John V. Frangioni, Cornelis J. H. van de Velde, Rutger-Jan Swijnenburg, Alexander L. Vahrmeijer

**Affiliations:** 1 Department of Surgery, University Medical Center, Albinusdreef 2, 2300 RC Leiden, The Netherlands; 2 Department of Radiology, University Medical Center, Leiden, The Netherlands; 3 Department of Medicine, Beth Israel Deaconess Medical Center, Boston, USA; 4 Department of Radiology, Beth Israel Deaconess Medical Center, Boston, USA

**Keywords:** Near-infrared fluorescence, Image-guided surgery, Indocyanine green, Hepatopancreatobiliary surgery

## Abstract

**Background:**

Improved imaging methods and surgical techniques have created a new era in hepatopancreatobiliary (HPB) surgery. Despite these developments, visual inspection, palpation, and intraoperative ultrasound remain the most utilized tools during surgery today. This is problematic, though, especially in laparoscopic HPB surgery, where palpation is not possible. Optical imaging using near-infrared (NIR) fluorescence can be used for the real-time assessment of both anatomy (e.g., sensitive detection and demarcation of tumours and vital structures) and function (e.g., assessment of luminal flow and tissue perfusion) during both open and minimally invasive surgeries.

**Methods:**

This article reviews the published literature related to preclinical development and clinical applications of NIR fluorescence imaging during HPB surgery.

**Results:**

NIR fluorescence imaging combines the use of otherwise invisible NIR fluorescent contrast agents and specially designed camera systems, which are capable of detecting these contrast agents during surgery. Unlike visible light, NIR fluorescent light can penetrate several millimetres through blood and living tissue, thus providing improved detectability. Applications of this technique during HPB surgery include tumour imaging in liver and pancreas, and real-time imaging of the biliary tree.

**Conclusions:**

NIR fluorescence imaging is a promising new technique that may someday improve surgical accuracy and lower complications.

## Introduction

Over the last few decades, imaging technologies such as ultrasonography (US), computed tomography (CT), magnetic resonance imaging (MRI), and positron emission tomography (PET) have become indispensable tools for preoperative planning in hepatopancreatobiliary (HPB) surgical procedures [1]. However, translating preoperative images to the surgical theatre still remains a major challenge. During HPB surgery, the surgeon mainly has to rely on visual inspection and palpation to discriminate between vital anatomical structures and, in case of a malignancy, between tumour and healthy tissue. Although in some cases intraoperative imaging modalities such as ultrasound or cholangiography can be applied [1, 2], irradical (R1) oncologic resections and iatrogenic surgical trauma are still major issues in HPB surgery.

Intraoperative imaging using near-infrared (NIR) fluorescence light is a novel technique that can provide real-time visualisation of tumour tissue and vital anatomical structures. It can also exploit physiological clearance of exogenous fluorescence contrast agents by the liver and biliary system to provide functional images of these structures. This makes NIR fluorescence imaging especially suitable for intraoperative imaging during HPB surgery.

This review describes the development, current applications, and future prospects of NIR fluorescence imaging for HPB surgery.

### Near-infrared fluorescence imaging

For intraoperative imaging, NIR fluorescent light has several advantages. The wavelength of NIR fluorescence lies between 700 and 900 nm, which is invisible to humans, and therefore does not alter the look of the surgical field. Further advantages of NIR light include high tissue penetration (up to 5 mm) and low autofluorescence. NIR fluorescence imaging is an inherently safe technique, as there is no ionizing radiation and no direct tissue contact. Image acquisition occurs within a few milliseconds, which allows the surgeon to operate under real-time image guidance. The introduction of minimally invasive techniques has increased the need for additional intraoperative imaging modalities. For example, NIR fluorescence imaging has been used to optimize sentinel lymph node dissection during robot-assisted laparoscopic surgery [3].

Several intraoperative NIR fluorescence camera systems have been developed for both open and laparoscopic surgery, some of which are commercially available. A description of the capacities of each of these systems is beyond the scope of this review, but is summarized by Gioux et al. [4]. State-of-the-art cameras can acquire a real-time overlay of the NIR channel with a normal colour channel [4–6]. This utility facilitates the surgeon with anatomical orientation within the surgical field, which is combined with the NIR fluorescent signal to allow image-guided surgery.

### NIR fluorescent contrast agents

In addition to an appropriate camera system, a NIR fluorescent contrast agent is also needed to visualise specific structures intended to be resected (e.g. tumour tissue) or to be spared (e.g. bile ducts). Such contrast agents contain a fluorescent moiety, which emits NIR fluorescent light after being excited with a NIR light source; and depending on the application, a targeting ligand that directs the fluorophore to the structure under study. Visualization of the tissue is based on the signal of the contrast agent in the region of interest relative to the background signal, known as signal-to-background ratio (SBR).

Indocyanine green (ICG) and methylene blue (MB) are the only NIR fluorophores that are registered with the Food and Drug Administration (FDA) and the European Medicines Agency (EMA) for clinical use, albeit for other indications. ICG has been registered for several decades to measure cardiac output, hepatic function, and ophthalmic perfusion. ICG emits fluorescent light at ≈800 nm, a property that has allowed its use in clinical NIR fluorescence imaging studies [7]. ICG is safe to use, as complications following administration are rare [8, 9]. For intraoperative imaging, ICG dose generally lies between 1 and 10 mg, but intravenous injection up to 25 mg has been reported to be safe [7]. Circulating ICG is cleared rapidly by the liver and almost exclusively excreted into the bile.

MB has been used for over 120 years for several medical applications [10]. It is used to treat sepsis and has been used at high dosages (>7.5 mg/kg) as a visible blue dye to stain the parathyroid glands during surgery [10–13]. When diluted to levels that are almost undetectable to the human eye, MB becomes a fluorophore emitting at ≈700 nm [14]. This phenomenon is known as “unquenching” [4]. At high dye concentration, fluorescence emission from MB is reabsorbed intermolecularly. When diluted, fluorescence emission becomes unquenched. At lower concentration, MB has a more favourable toxicity profile, although when used at high dosages (>7.5 mg/kg) serious adverse reactions have been reported, particularly in patients taking serotonin reuptake inhibitors (SSRI’s) [15]. MB is cleared equally by both the liver and kidney, permitting imaging of both bile ducts and ureters [16, 17].

The chemical structures of both ICG and MB do not allow these agents to be conjugated to tissue-specific ligands. As such, they are nonspecific NIR contrast agents. To permit targeted imaging, such as tumour imaging, novel NIR fluorescent probes are being developed, as recently reviewed by Luo et al. [18]. These fluorophores can be conjugated to a tumour-specific ligand to target tumour cells [19]. For instance, they can be conjugated to tumour-specific antibodies [20], nanobodies [21], small peptides, [20] or they can be activated by enzymatic cleavage in order to become fluorescent [22]. A major drawback in the development of new NIR fluorescence contrast agents is that each fluorophore-target conjugate needs separate regulatory approval, which is an expensive and time-consuming process.

The following section of this review will focus on the different applications of NIR fluorescence imaging during HPB surgery.

### Tumour imaging

#### Liver cancer and colorectal metastases

Liver cancer accounted for an estimated 748,300 new cases and 695,900 cancer deaths worldwide in 2008 [23]. Colorectal cancer is the second cause of cancer death worldwide [24] and the survival of colorectal carcinoma patients mainly depends on the occurrence of distant metastases, which occur most frequently in the liver. Improvements in surgical techniques, preoperative imaging, and neoadjuvant chemotherapy have led to an increase in the percentage of patients eligible for resection of liver tumours. For intraoperative identification of liver tumours, visual inspection, palpation, and intraoperative ultrasound (IOUS) are used routinely. Still, recurrence rates of colorectal liver metastases (CLM) vary between 11 and 37.5 %, of which 65–85 % appear within 2 years following surgery [25–29]. A possible explanation for this high recurrence rate is the presence of small malignant lesions that are missed by current preoperative and intraoperative detection methods.

**Fig. 1:**
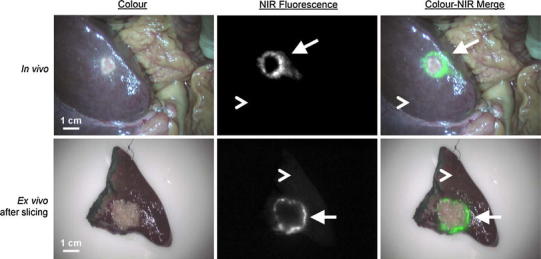
NIR fluorescence imaging of colorectal liver metastases using the Mini-FLARE imaging system: a colorectal liver metastasis (*arrow*) is clearly identified by a NIR fluorescent rim around the tumour in vivo (*top row*), 24 h after injection of 10-mg ICG. Normal liver tissue (*arrowhead*) shows minimal retention of ICG. After resection and slicing of the specimen, the rim around the tumour is better visualized ex vivo (*bottom row*) (van der Vorst et al., unpublished data)

NIR fluorescence imaging using ICG is a promising technique to assist in the identification of primary liver tumours and CLM [30–36] during surgery. Due to the hampered visibility and inability to palpate the liver surface during laparoscopy, NIR fluorescence imaging could also be of great value during minimally invasive liver surgery. It has been shown that ICG passively accumulates in hepatocellular carcinomas (HCC) and in a rim around CLM when intravenously administered 1–14 days before surgery [30] (Fig. 1 also shows an example of this phenomenon). However, it is important to realise that accumulation of ICG in liver tumours is based the pharmacokinetic clearance of ICG and is therefore most likely subject to liver perfusion, ICG clearance and bile drainage.

**Fig. 2:**
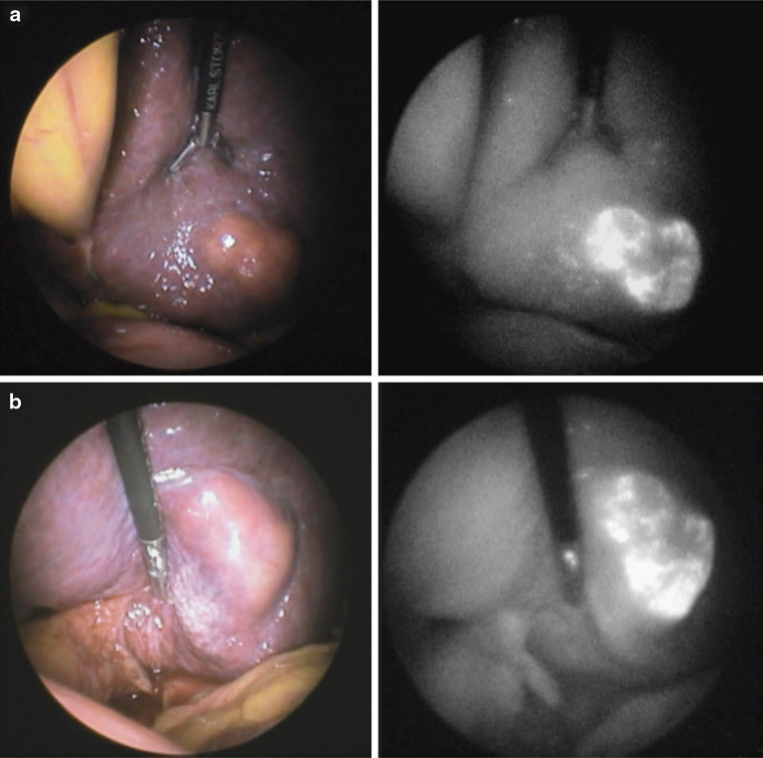
Laparoscopic NIR fluorescence imaging of a hepatocellular carcinoma: **a** Colour image (*left*) and fluorescent image (*right*) of the visceral surface of the left liver before mobilization. Fluorescent imaging clearly delineated the hepatocellular carcinoma located in segment II, with the surrounding structures. **b** The left liver was fully mobilized and the lesser omentum was sectioned, using NIR fluorescence imaging to confirm the appropriateness of the resection margin of the tumour (adapted from Ishizawa et al. [31] and reprinted with permission from John Wiley & Sons, Inc)

Several clinical studies describe the use of NIR fluorescence imaging to visualise HCC (Table 1) [31, 32, 34, 37]. In these studies combined, a total 32 patients were included. In all patients 0.5-mg/kg ICG was administered intravenously 1–8 days before surgery. All superficially located tumours could be clearly identified using NIR fluorescence. However, due to the limited penetration depth tumours located deeper than approximately 5 mm under the liver capsule could only be identified after resection and sectioning. Importantly, Gotoh et al. [32] reported identification of additional occult HCC nodules in 4 out of 10 cases that were not detected by use of any preoperative examinations. However, Ishizawa et al. [30] reported in a group of 63 HCCs 5 false positive nodules. The first successful case of liver tumour imaging during laparoscopic surgery in a patient suffering from a HCC was recently reported (Fig. 2) [31].

**Table 1:** Identification of primary liver tumours and liver metastases

Study	Years	Number of patients	Cancer type	Imaging system	Dose of ICG	Injection site	Time between injection and imaging	Intraoperative IR (tumours)	Additional metastases identified
Gotoh	2009	10	HCC	Photo dynamic eye	0.5 mg/kg	i.v.	1–8 days	10/10	+
Harada	2009	3	ICC (*n* = 2) and CLM (*n* = 1)	Photo dynamic eye	0.5 mg/kg	i.v.	4 days [1, 2] and 2 days (3)	3/3^a^	−
Ishizawa	2009	49^b^	HCC (*n* = 37) and CLM (*n* = 12)	Photo dynamic eye	0.5 mg/kg	i.v.	1–7 days for HCC and 1–14 days for CLM	21/41 HCCs and 16/16CLM^c^	+
Ishizawa	2010	1	HCC	Laparoscope, Hamamatsu	0.5 mg/kg	i.v.	5 days	1/1	−
Kasuya	2010	1	CLM	Photo dynamic eye	500 μl mixed with ethanol	Locally injected	NA	NA	−
Kawaguchi	2011	1	HCC	HyperEye	0.5 mg/kg	i.v.	3 days	1/1	−
Uchiyama	2010	32	CLM	Photo dynamic eye	0.5 mg/kg	i.v.	<2 weeks	NA	+
Van der vorst et al.	2011	22	CLM	Mini-FLARE	10 and 20 mg	i.v.	24 and 48 h	40/43	+
Yokoyama	2011	49	Pancreatic cancer metastases	Photo dynamic eye	25 mg	i.v.	1 day	NA	+

*HCC* hepatocellular carcinoma, *CLM* colorectal liver metastases, *ICC* intrahepatic cholangiocarcinoma, *NA* not available, *IR* identification rate, *i.v.* intravenous, *i.b.* intrabilary

^a^Fluorescent imaging clearly identified the regions of the liver with cholestasis caused by tumour invasion. The tumour itself was not fluorescent

^b^From 49 patients, 26 patients (20 with HCC and 6 with CLM) underwent fluorescent imaging during surgery

^c^Identification rate of the 26 patients that where examined during surgery

A number of clinical studies have described the use of ICG for visualization of CLM (Table 1). These studies combined included a total of 68 patients [30, 33, 35, 36]. Ishizawa et al. [30] described demarcation of both HCC and CLM using intravenous administration of ICG. With intraoperative imaging, 21 of the 41 HCCs were detected and all CLM (which were all superficially located). After resection and sectioning, all liver tumours could be identified by both gross examination and fluorescence imaging, resulting in a sensitivity and positive predictive value of 100 % of this technique on surgical specimens ex vivo. ICG (0.5 mg/kg) was injected ranging from 1 to 14 days prior to surgery. The macroscopically confirmed CLM metastases were identified by a rim fluorescent pattern surrounding the lesion. It was hypothesized that this could be due to compromised biliary excretion by the surrounding noncancerous liver tissues, which are compressed by the tumour. It has also been reported that in patients with a poor ICG retention rate (i.e. decreased clearance function of the liver), the fluorescence signal of the noncancerous liver parenchyma was higher. This makes it more challenging to obtain an adequate tumour to liver contrast, in particular in case of cirrhosis, steatosis or chemotherapy pretreatment.

Uchiyama et al. [33] showed similar results in 32 CLM patients. The use of intraoperative ultrasound in combination with NIR fluorescence improved the diagnostic sensitivity compared to the use of CT and MRI from 46/52 (88.5 %) to 51/52 metastases (98.1 %, *P* = 0.050). With the use of NIR fluorescence, 2 additional malignant lesions could be identified that otherwise would have been missed. Van der Vorst et al. performed a NIR fluorescence study for the identification of CLM metastases using ICG in 22 consecutive patients (manuscript under review) and found similar results; additionally, Van der Vorst et al. optimized ICG dosing and timing.

Another study investigated the potential of this technique to detect small hepatic metastases in pancreatic cancer patients. Consecutive patients (*n* = 49) with pancreatic cancer who were eligible for surgical resection with curative intent were examined. In these patients, no hepatic tumours were identified on either preoperative imaging, intraoperative inspection, or US. Following injection of 25-mg ICG [38], NIR fluorescence imaging detected abnormal hepatic lesions in 13 patients. In 8 out of 13 lesions, micrometastases were found (8 out of 49 included patients: 16 %). This suggests that NIR fluorescence imaging has the potential to detect hepatic pancreatic cancer micrometastases that were not found using currently existing imaging techniques, and could help surgeons select patients for either curative or palliative surgery.

It has also been demonstrated that NIR fluorescence imaging can be used to identify hepatic segments and subsegments for anatomical hepatic resection [39]. Aoki et al. performed a study in 35 patients with malignant liver disease who underwent hepatectomy. ICG (5 mg) was administered into the portal vein branch, and subsequently, NIR images were obtained. Stained subsegments and segments of the liver were identified in 33 of the 35 patients. The procedure was unsuccessful in 2 patients; however, this was probably due to difficulties with ICG administration. Segmental mapping with NIR fluorescence seems a promising technique to assist the surgeon during anatomic resections of the liver. Uchiyama et al. [40] combined intraoperative fluorescent imaging with contrast-enhanced ultrasonography in 22 patients for intraoperative liver resection. Following portal pedicle ligation, 0.5 mg/kg ICG was injected intravenously. Subsequently the negative-brightness area of the liver without blood flow was detected on the liver surface using the PDE system. Demarcation line of the liver surface after the portal pedicle ligation was apparent in 17 patients; however, the resection line using PDE was clearly detected in all patients. Segmental mapping with NIR fluorescence seems a promising technique to assist the surgeon during anatomic resections of the liver.

Preoperative administration of ICG was also used to visualise cholestatic liver parenchyma during surgery [35]. In a pilot study, 2 patients diagnosed with intrahepatic cholangiocarcinoma and 1 patient with CLM metastases and a suspected bile duct thrombus were included. NIR fluorescent imaging could clearly demarcate the cholestatic regions on the surface of the liver caused by bile duct tumour invasion or thrombi in all 3 patients [35]. One study reported the use of percutaneous injection of ICG mixed with ethanol (dilution: 1:100) for hepatic tumour resection [36]. Demarcation of the target area with ICG-ethanol using real-time virtual sonography and fluorescence based resection was successful in 1 patient. The technique could potentially be helpful for resection of hepatic tumours which cannot be identified by routine ultrasonography.

Several preclinical studies assessed the use of NIR probes for intraoperative detection of liver tumours. To optimize dose and timing of ICG for detection of CLM, van der Vorst et al. [41] performed a preclinical study in 18 rats bearing CLM. This study reported that the optimal timing of ICG administration was 72 h before surgery and that the optimal dose was 0.25-mg/kg ICG. Tumour-to-liver ratios of approximately 3 were reported in this study. In an attempt to improve tumour-to-liver ratios, NIR fluorescence imaging using lactosome nanocarriers labelled with ICG in a hepatocellular carcinoma (HCC) bearing mouse model was performed [42]. The ability to visualize liver tumours was based on the enhanced permeation and retention (EPR) effect of the lactosome. EPR is caused by leaky blood vessels and hampered lymphatic drainage in the tumour [43, 44]. Tumour targeted NIR fluorescence imaging of CLM was shown in an orthotopic rat model using an integrin α_*v*_β_3_ targeting NIR probe. Integrin α_*v*_β_3_ plays a key role in tumour angiogenesis, tumour cell migration and is over expressed in various cancer types [45]. All CLM could be clearly visualized and clinically relevant tumour-to-liver ratios of approximately 2 were reported in this study [20].

In conclusion, several studies showed that NIR fluorescence imaging is feasible for liver tumour imaging and segmental demarcation. Due to passive accumulation of ICG, superficial liver metastases can be identified with high sensitivity. A pitfall of this technique is the limited tissue penetration of NIR light, which is approximately 5 mm. This can be problematic for tumours located deeper beneath the liver capsule. Nevertheless, this technique could be of great value for the detection of small superficial lesions, and newer optical techniques are being developed to interrogate 1 cm or more.

#### Pancreatic tumours

Pancreatic adenocarcinoma is the fourth leading cause of cancer-related death in the Western world [46]. The overall 5-year survival rate is <5 %, showing no substantial improvement over the past 30 years [46]. The main prognostic factor in pancreatic cancer patients that are eligible for resection is the involvement of tumour-positive resection margins. Reported median survival rates after a R0 resection for pancreatic adenocarcinoma are twice as high as compared to a R1 resection [47]. Irradical resection of pancreatic cancers still occurs in 34.7–42 % of patients [48–50]. Advances in preoperative imaging modalities have improved the ability to estimate respectability and to differentiate pancreatic carcinoma from other pancreatic diseases [1]. However, as mentioned before, translating these images to the operating room remains challenging.

NIR fluorescence imaging offers new opportunities for intraoperative pancreatic tumour visualization. Thus far, only 1 clinical study has attempted to visualize pancreatic tumours using this technique. Based on previous studies that showed that solid tumour accumulation is achievable using a nontargeted probe based on EPR effect, Hutteman et al. attempted to use this concept for pancreatic cancer imaging with ICG. However, the results were disappointing [51]. After intravenous injection of ICG, a pancreatic tumour could be visualized in only 1 of 8 patients. This could possibly be explained by the lower perfusion of adenocarcinomas of pancreas in comparison with healthy pancreatic tissue, which might decrease availability of ICG for a potential EPR effect of the tumour [52, 53].

Several preclinical studies have shown adequate tumour identification in different pancreatic tumour animal models using NIR fluorescence. Von Bursin et al. showed the use of a tumour targeted protease activatable NIR probe to obtain tumour identification in a mouse model of early-stage pancreatic cancer [54]. Using this technique it was possible to discriminate between normal pancreatic tissue, inflammation, and early-stage malignancy. Tran Cao et al. [55] described successful fluorescence laparoscopy to image green fluorescent protein (GFP)-expressing tumours in an orthotopic mouse model of human pancreatic adenocarcinoma using a GFP-specific NIR fluorescent probe. They argued that fluorescence laparoscopy of tumours labelled with GFP or fluorescent antibodies could be used for diagnosis and staging of pancreatic cancer in the future.

NIR fluorescence imaging of neuroendocrine pancreatic tumours have also been investigated in animal models. Winer et al. [56] showed the ability to clearly visualise pancreatic insulinomas using MB. Intravenous administration of MB permitted high-sensitivity, real-time localization of primary, multicentric, and metastatic insulinoma and permitted differentiation among tumour, normal pancreas, and other abdominal structures. At doses >1 mg/kg, high SBRs up to 3 were observed for up to 1 h after administration [56]. MB is a phenothiazine derivative that acts as a perfusion tracer. As such, it is likely that hypervascular tumours, such as insulinomas can be visualised using MB, although the complete mechanism of this phenomenon still remains unclear.

In conclusion, although the identification of pancreatic tumours was successful in preclinical models, clinical NIR fluorescence imaging of these tumours is still to be accomplished. The development of clinical available tumour specific contrast agents seems imperative.

### Bile imaging

#### Bile duct imaging

During laparoscopic cholecystectomy, common bile duct (CBD) injury is a rare but severe complication with a postoperative mortality of 11 % [57]. It has been stated that intraoperative cholangiography can reduce the risk of CBD injury from 0.58 to 0.39 % [57]. However, recent literature questions the advantage of routine cholangiography [58]. Furthermore, intraoperative cholangiography is time consuming and the introduction of a catheter into the CBD can induce leakage [59]. Laparoscopic ultrasonography is a less invasive and safe method to identify CBD stones and anatomical abnormalities, but its use has not been deployed broadly.

**Fig. 3:**
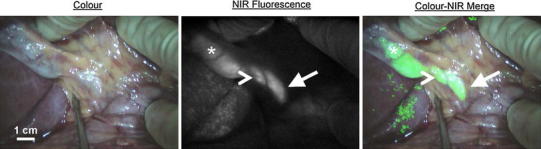
NIR fluorescence imaging of the bile duct during liver surgery: colour video (*left panel*), NIR fluorescence (*middle panel*), and a colour-NIR overlay (*right panel*) of intraoperative imaging of the cystic duct (*arrowhead*) and common bile duct (*arrow*) in a patient who underwent liver resection for colorectal metastases, 24 h after administration of 10-mg ICG. The *asterisk* indicates the position of the gallbladder

NIR fluorescence imaging provides new opportunities for safe exploration of biliary anatomy and function during hepatobiliary surgery. The most common method is to intravenously inject a fluorophore like ICG, which is excreted into the bile, resulting in fluorescence cholangiography (Fig. 3). Some studies also reported retrograde injection of an NIR probe into the bile duct, however, introduction of a catheter could result in iatrogenous damage.

A total of 9 clinical studies report the use of ICG as a NIR fluorescent dye for bile duct imaging after intravenous administration (Table 2) [34, 37, 60–66]. Seventy-five patients undergoing laparoscopic and 27 patients undergoing open bile duct imaging were included. In general, ICG was injected intravenously 30 min prior to surgery, and most studies used a dose of 2.5 mg (with the exception of one study that reported the use of 12.5 mg ICG [63] and one study that reported the use of 5 and 10 mg ICG [51]). Using fluorescence cholangiography during laparoscopic cholecystectomy, Aoki et al. [63] showed identification of the CBD and cystic duct in 10 of 14 patients. The authors stated that unsuccessful identification of the bile ducts was due to obesity. In these patients, fatty tissue surrounding the bile ducts limited visualization.

**Table 2:** Bile duct imaging using NIR fluorescence

Study	Years	Number of patients	Procedure	Imaging system	Dose of ICG	Injection site	Timing of injection	IR of bile ducts (patients)
Aoki et al.	2009	14	LC	Laparoscope, Hamamatsu	12.5 mg	i.v.	30 min preoperative	10/14
Hutteman et al.	2011	8	Pancreaticoduodenectomy	Mini-FLARE	5 and 10 mg	i.v.	During OR	8/8
Mitsuhashi et al.	2008	5	OC	Photo dynamic eye	2.5 mg	i.v.	30 min preoperative	5/5
Mizuno et al.	2010	1	Cholangiography for donor hepatectomy	Photo dynamic eye	0.025 mg/ml^a^	i.b.	During OR	1/1
Ishizawa et al.	2008	23 (13/10)	Hepatectomy (*n* = 13) and cholecystectomy (*n* = 10)	Photo dynamic eye	0.025 mg/ml^a^ and 2.5 mg	i.b. and i.v..	During OR and 30 min preoperative	i.b. = 13/13, i.v. = 10/10
Ishizawa et al.	2009	1	LC	Laparoscope, Hamamatsu	2.5 mg	i.v.	2 h preoperative	1/1
Ishizawa et al.	2010	52	LC	Laparoscope, Hamamatsu	2.5 mg	i.v.	30 min preoperative	52/52
Ishizawa et al.	2011	7	Single incision LC	Laparoscope, Hamamatsu	2.5 mg	i.v.	After intubation on OR	7/7
Kawaguchi et al.	2011	2	Liver transplantation and partial liver resection for HCC	HyperEye	0.025 mg/ml^a^ and 2.5 mg	i.b. and i.v.	Before division of hepatic duct and after liver resection	2/2
Tagaya et al.	2010	12 (8/4)	LC (*n* = 8) and open OC (*n* = 4)	Prototype laparoscope and photo dynamic eye	2.5 mg	i.v.	1–2 h preoperative	12/12

*IR* identification rate, *LC* laparoscopic cholecystectomy, *OC* open cholecystectomy, *i.v.* intravenous, *i.b.* intrabilary, *HCC* hepatocellular carcinoma

^a^Amount of ICG not available

For dose optimization and biodistribution purposes, Hutteman et al. visualized the CBD and cholangiojejunostomy during open pancreaticoduodenectomy after intravenous injection of ICG in 8 patients. Between 10 and 90 min after administration of 5- and 10-mg ICG, the CBD could clearly be identified by NIR fluorescence imaging in all patients. No difference was observed between the 5- mg and 10-mg groups (*P* = 0.849). Highest SBRs were found between 30 and 90 min postinjection, with a maximum mean SBR of 6.2 ± 1.3 at 60 min postinjection [51].

A total of 5 preclinical studies reported the use of NIR fluorescence imaging to visualize the biliary anatomy in animal models [16, 64, 65, 67, 68]. Four of these studies used ICG. After surgical exposure and intravenous injection of ICG, bile duct imaging was successful in all experiments. Due to hepatic clearance of ICG, timing of ICG injection is crucial to minimize background signal of the liver. One study tested both ICG and IRDye^®^ 800CW fluorophore (LI-COR, biosciences, Lincoln, Nebraska) [67]. A single intravenous injection of IRDye^®^ CW800-Carboxylate at doses >0.0015 mmol/kg resulted in a high SBR (±3) of the CBD for at least 30 min post-injection, with a dose of 0.0075 mmol/kg being optimal. Due to the quick excretion of IRDye^®^ 800CW, liver background signal was low, which can be an advantage over ICG. Another study used MB and ICG for biliary imaging in pigs [16]. Bile duct imaging was successful in all animals. Contrast-to-background ratio for MB and ICG were roughly equivalent at later time points after injection. The use of ICG’s fluorescence at 800 nm has the advantage of lower autofluorescence from the surrounding bowel and tissue, but the disadvantage of longer retention in the liver resulting in higher background. As a consequence, ICG required a long lag time (>90 min) before adequate contrast could be observed relative to the liver. The ICG signal lasted for up to 240-min postinjection. MB signal intensity in the CBD became visible within minutes, and remained adequate for imaging for up to 120 min. Advantages of MB include low liver uptake and rapid excretion into bile. Disadvantages include a 700-nm rather than an 800-nm emission and a relatively low quantum yield.

A recent study tested a novel NIR lipophilic probe VM674 (VisEn Medical, Bedford, MA). This probe allows rapid biliary excretion after intravenous administration [68]. It was tested in mice with chronic biliary obstruction, acute biliary obstruction, bile duct perforation and choledocholithiasis [64]. Bile duct visualisation was successful after intravenous administration of 10 nmol and it was feasible to visualize bile duct obstruction, perforation, and choledocholithiasis. Optimal SBR of 6.4 ± 0.8 was observed after 25 min post intravenous injection and a clear signal was visible for up to 60 min.

In conclusion, to perform fluorescence cholangiography, dose and timing of contrast agent administration is crucial. According to the available clinical literature, the most optimal parameters to perform clinical NIR fluorescence bile duct imaging are the administration of 2.5 mg of ICG approximately 30 min before surgery.

#### Bile leakage imaging

Bile leakage is a serious complication after HPB surgery [69]. There is a strong association between bile leakage after hepatic resection and a high risk for liver failure and surgical mortality [69]. Early detection of bile leakage could potentially prevent late complications, such as liver failure. To identify bile leakage during surgery, different approaches have been described [70, 71]. A randomized trail in 103 patients investigated the value of intrabiliary injection of saline to identify bile leakage, but found no significant difference. [70] A trial that investigated injection of a fat emulsion reported a reduction in bile leakage; however, complete prevention of bile leakage was not achieved [71].

Two clinical studies have demonstrated the use of NIR fluorescence for bile leakage identification after hepatic resection [72, 73]. ICG was injected intrabiliary after hepatic resection through a transcystic catheter. The CBD was clamped distal to the cystic duct and fluorescent imaging was performed. In a group of 102 patients undergoing hepatic resection, 5 patients developed postoperative bile leakage in the control group versus no patients in the group in which NIR fluorescence was used (*P* = 0.019) [72]. This suggests an additive value of NIR fluorescence imaging for intraoperative detection of bile leakage.

## Discussion and future prospects

Image-guided HPB surgery using invisible NIR fluorescent light has been intensely explored over a relatively short time. The technique has shown potential to improve intraoperative identification and demarcation of tumours and vital structures. It could potentially be a useful tool to reduce the number of positive resection margins and to prevent re-interventions. Applications include the identification and demarcation of HPB tumours, identification of pancreatic liver metastases during pancreatic surgery, real-time bile duct imaging and bile leakage detection. In addition, it is also possible to acquire functional images of the liver and bilary system by physiological clearance of exogenous fluorescence contrast agents that are almost completely cleared by the liver (i.e. ICG).

The clinical availability of ICG and MB allowed NIR fluorescence image-guided surgery to be introduced into clinical trials. However, for tumour imaging to make the next step into development and acceptance of this new technique, tumour targeted contrast agents will be essential. As described in this review, many preclinical studies showed promising results using tumour specific probes. However, the most important drawback in the clinical implementation of these probes is that each probe has to go through a separate expensive and time-consuming regulatory approval processes.

Development of new imaging systems will offer new prospects for image-guided surgery, as most current imaging systems are still in the experimental phase. Major improvements are to be expected concerning image resolution, sensitivity, and fluorescence-guided laparoscopy. The use of laparoscopy in HPB surgery is steadily increasing, which makes it a particularly interesting field for the development and improvement of laparoscopic fluorescence imaging systems. To date, quantification in optical imaging remains challenging. As tissue absorbance and probe concentration at the site of the tumour will vary among patients, the contrast between target tissue (tumours, vital structures or lymph nodes) and surrounding tissue is important and will be the basis of clinical decision making. Current research is focused on new methods to improve signal quantification, for example by means of time domain imaging and spatial frequency domain imaging [74, 75].

One of the major challenges of NIR fluorescence imaging is its limited penetration depth. To date, the available literature reports a penetration depth ranging from several millimetres to, at most and in rare circumstances, 1 cm. A future goal is the development of novel fluorophores and camera systems to improve these numbers.

Finally, to validate the use of NIR fluorescence imaging, and more importantly to quantify patient benefit, large clinical trials will be essential. Despite the very promising results already obtained, the next decade will define the true efficacy of NIR fluorescence imaging during HPB surgery.
